# Dynamic changes of mitochondrial homeostasis and immune-inflammatory signaling during hemorrhagic shock resuscitation associated ischemia–reperfusion injury

**DOI:** 10.3389/fimmu.2026.1910044

**Published:** 2026-10-13

**Authors:** Yuting Zhou, Xiaoyu Zhang, Huifang Gao, Nan Ji, Bixi Li

**Affiliations:** 1 Department of Anesthesiology, General Hospital of Central Theater Command of People's Liberation Army (PLA) Graduate Joint Training Base, School of Medicine, Wuhan University of Science and Technology, Wuhan, Hubei, China; 2 General Hospital Base of Central Theater Command of People’s Liberation Army, Hubei University of Medicine, Wuhan, China; 3 Department of Anesthesiology, General Hospital of Central Theater Command of People's Liberation Army (PLA), Wuhan, Hubei, China

**Keywords:** damage-associated molecular patterns, fluid resuscitation, ischemia-reperfusion injury, mitochondrial homeostasis, traumatic hemorrhagic shock

## Abstract

Traumatic hemorrhagic shock is a common and life-threatening condition following severe trauma, characterized by reduced effective circulating blood volume and insufficient tissue perfusion, ultimately leading to an imbalance between oxygen supply and demand as well as progressive multi-organ dysfunction. Fluid resuscitation is essential for restoring hemodynamic stability; however, it may simultaneously trigger or aggravate ischemia-reperfusion injury (IRI). Emerging evidence suggests that disruption of mitochondrial homeostasis represents not only a critical cellular basis underlying organ injury during hemorrhagic shock resuscitation, but also an important driver of immune activation and inflammatory amplification. At the level of mitochondrial homeostasis regulation, the ischemia-reperfusion process exhibits distinct phasic alterations across the ischemic phase, early reperfusion, and late reperfusion stages. These dynamic changes are primarily characterized by metabolic reprogramming, excessive oxidative stress, mitochondrial dynamics imbalance, impaired mitophagy, loss of mitochondrial membrane integrity, and the release of mitochondria-derived damage-associated molecular patterns (DAMPs). Collectively, these events contribute to the initiation and progression of IRI and further promote dysregulated inflammatory responses. This review summarizes the phasic alterations of mitochondrial homeostasis and their interactions with immune-inflammatory pathways during hemorrhagic shock resuscitation. Understanding these temporal mechanisms may provide a rationale for stage-specific mitochondrial-targeted therapeutic strategies aimed at improving organ protection and modulating dysregulated immune-inflammatory responses.

## 1 Introduction

Traumatic hemorrhagic shock is a life-threatening condition resulting from severe trauma and is characterized by a marked reduction in effective circulating blood volume, decreased cardiac output, and impaired microcirculatory perfusion. These alterations disrupt the balance between tissue oxygen delivery and metabolic demand, ultimately leading to cellular metabolic dysfunction, progressive organ injury, and even multiple organ dysfunction syndrome (1).

Fluid resuscitation is a fundamental therapeutic strategy for hemorrhagic shock that aims to restore circulating blood volume, improve hemodynamic stability, and maintain the perfusion of vital organs. Consequently, it is widely applied during the early phase of shock management (1, 2). However, accumulating evidence indicates that rapid or excessive fluid resuscitation, although capable of promptly restoring tissue perfusion, may also rapidly transition ischemic tissues to reperfusion, thereby triggering systemic IRI (3). The choice of resuscitation fluid may further influence the extent and course of cellular recovery following reperfusion. Traditional crystalloid or colloid-based resuscitation primarily restores intravascular volume, whereas contemporary blood-based strategies, including red blood cells and plasma, additionally aim to restore oxygen-carrying capacity and hemostatic function (4, 5). These differences may affect tissue oxygen delivery, microcirculatory perfusion, inflammation, and edema, which are closely linked to mitochondrial function during reperfusion (6). Nevertheless, direct evidence regarding how different resuscitation strategies affect mitochondrial homeostasis and its subsequent recovery remains limited. Therefore, the choice of resuscitation strategy may represent an important contextual factor when considering the dynamic changes in mitochondrial homeostasis during reperfusion.

Mitochondrial homeostasis refers to the integrity of the mitochondrial network and the maintenance of its physiological functions (7). Growing evidence indicates that IRI is closely associated with the disruption of mitochondrial homeostasis. These alterations not only aggravate local tissue injury but also contribute to distant organ damage and systemic inflammatory responses through excessive oxidative stress and the release of inflammatory mediators (8).

During hemorrhagic shock resuscitation, mitochondrial homeostasis undergoes distinct dynamic changes across three major stages: the ischemic phase, early reperfusion, and late reperfusion. These changes involve multiple interconnected mechanisms, including impaired mitochondrial energy metabolism, excessive oxidative stress, dysregulation of mitochondrial dynamics, defective mitophagy, mitochondrial permeability transition pore (mPTP) opening, and the release of mitochondria-derived damage-associated molecular patterns. Importantly, these processes may overlap temporally and interact with one another, with their relative contributions varying across different stages of ischemia-reperfusion (**Figure 1**).

**Figure 1:**
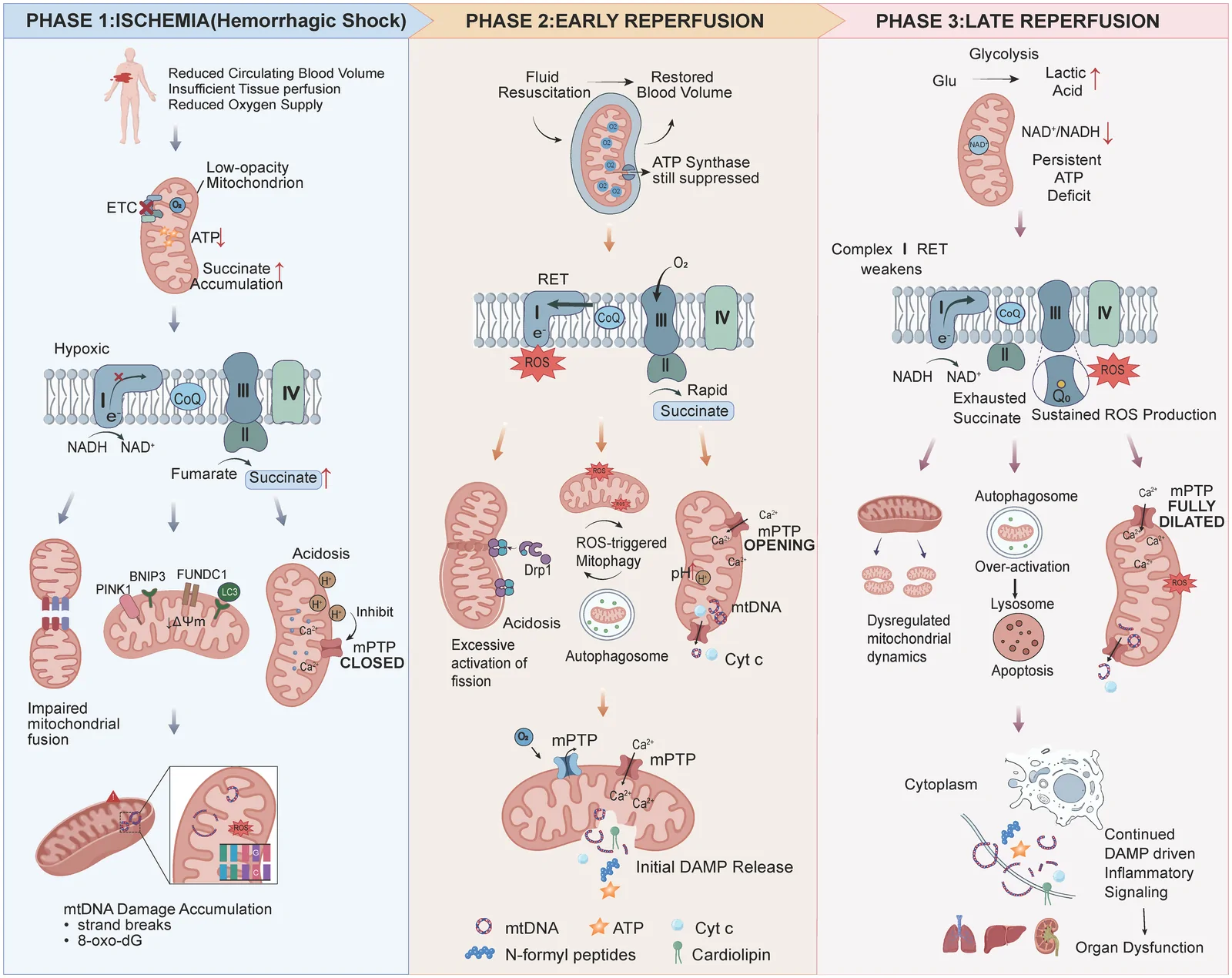
Dynamic changes of mitochondrial homeostasis during hemorrhagic shock resuscitation associated ischemia–reperfusion injury. During the ischemic phase, oxygen deprivation suppresses electron transport chain (ETC) activity and oxidative phosphorylation, leading to adenosine triphosphate (ATP) depletion, succinate accumulation, impaired mitochondrial fusion, activation of mitophagy-related pathways, and progressive mtDNA damage. During the early reperfusion phase, restoration of oxygen delivery rapidly oxidizes accumulated succinate, driving reverse electron transport (RET) and a burst of reactive oxygen species (ROS) generation. Excessive ROS production and Ca^2+^ overload promote mitochondrial fragmentation, mitophagy activation, and mPTP opening, resulting in initial release of DAMPs associated with mitochondrial injury, including mtDNA, ATP, N formyl peptides, cardiolipin and Cytochrome c (Cyt c). During the late reperfusion phase, persistent metabolic dysfunction is characterized by enhanced glycolysis, lactate accumulation, a reduced NAD^+^/NADH ratio, and sustained ATP deficiency. Persistent mitochondrial dysfunction sustains ROS production, dysregulated mitochondrial dynamics, excessive autophagic activity, apoptosis, and prolonged mPTP opening, leading to sustained DAMP driven inflammatory signaling and subsequent organ dysfunction.

Current studies on IRI following hemorrhagic shock resuscitation have largely focused on individual stages or isolated mechanisms, such as oxidative stress, mitochondrial dysfunction, and inflammatory responses. However, a systematic understanding of the dynamic changes in mitochondrial homeostasis across different stages and the mechanistic interplay among these processes remains lacking. In particular, a comprehensive temporal framework integrating the progression of mitochondrial dysfunction, mitochondrial DAMP release, and subsequent immune-inflammatory signaling remains limited.

Accordingly, this review adopts the ischemic phase, early reperfusion, and late reperfusion as a temporal framework to systematically summarize the stage-specific changes in mitochondrial homeostasis and the associated molecular mechanisms. Furthermore, the interactions between mitochondrial dysfunction and immune-inflammatory signaling are discussed to provide insights into organ protection and identify potential therapeutic targets for mitochondria-targeted and immunomodulatory strategies following hemorrhagic shock resuscitation.

## 2 Mitochondrial energy metabolic dysfunction

Mitochondrial energy metabolism dysfunction is a hallmark of the ischemic phase and represents an early manifestation of mitochondrial functional impairment, thereby laying the foundation for subsequent structural damage. During traumatic hemorrhagic shock, acute blood loss results in a rapid decline in effective circulating blood volume and inadequate tissue perfusion. Although fluid resuscitation can partially restore circulating volume, tissue perfusion and oxygen delivery often remain insufficient because of persistent microcirculatory dysfunction and blood flow redistribution (9). Sustained hypoperfusion exposes tissues to a prolonged hypoxic microenvironment, which directly impairs mitochondrial oxidative phosphorylation and subsequently disrupts cellular energy metabolism.

As the primary organelles responsible for cellular energy production, mitochondria rely on the integrity of the electron transport chain (ETC) to maintain efficient oxidative phosphorylation. Under ischemic conditions, ETC impairment reduces oxidative phosphorylation efficiency and adenosine triphosphate (ATP) production, ultimately leading to insufficient cellular energy supply and progressive cellular and organ dysfunction (10). Notably, during hemorrhagic shock and subsequent resuscitation, cellular energy metabolism is markedly suppressed, and this metabolic disturbance may persist even after apparent hemodynamic recovery, suggesting that mitochondrial dysfunction may be sustained despite the restoration of systemic circulation.

During the ischemic phase, acute blood loss results in inadequate tissue perfusion and reduced oxygen delivery, thereby impairing mitochondrial oxidative phosphorylation and progressively disrupting cellular energy metabolism. In a rat model of hemorrhagic shock, Villarroel et al. (6) demonstrated that mitochondrial basal respiration, maximal respiration, and ATP-linked respiration in peripheral blood mononuclear cells were all markedly decreased and were accompanied by a reduction in mitochondrial membrane potential (ΔΨm). These findings indicate substantial impairment of mitochondrial oxidative phosphorylation and reduced ATP production under ischemic conditions.

During the early reperfusion phase, although fluid resuscitation can partially restore circulating blood volume and systemic hemodynamics, mitochondrial function does not recover concomitantly with systemic hemodynamic restoration. Warren et al. (11) reported in a standardized rat model of controlled hemorrhagic shock that mitochondrial respiration and ATP production capacity remained incompletely restored following fluid resuscitation. These findings suggest that despite the apparent improvement in hemodynamic parameters, mitochondrial energy metabolism remains suppressed during the early reperfusion phase.

During the late reperfusion phase, cellular metabolism undergoes persistent metabolic reprogramming, primarily characterized by sustained dependence on glycolysis and incomplete recovery of mitochondrial oxidative phosphorylation following resuscitation. Although glycolysis can partially compensate for ATP production during the early reperfusion phase, its energy conversion efficiency is substantially lower than that of mitochondrial oxidative phosphorylation. Consequently, overall ATP availability remains insufficient throughout the late reperfusion phase. In a rat model of hemorrhagic shock employing different resuscitation strategies, D’Alessandro et al. (12) demonstrated that lactate levels increased markedly following hemorrhagic shock. Conventional crystalloid resuscitation failed to effectively reverse this metabolic disturbance, whereas plasma-first resuscitation only partially reduced lactate accumulation without fully restoring metabolic homeostasis. These findings suggest that persistent metabolic abnormalities remain despite the re-establishment of systemic circulation. Consistently, a metabolomics study by Slaughter et al. (13) revealed that, in a tissue injury–hemorrhagic shock–resuscitation model, glycolytic intermediates continued to accumulate abnormally in multiple tissues during the late reperfusion phase, indicating sustained activation of the glycolytic pathway. Furthermore, Rao et al. (14) found that even after administration of liposome-encapsulated hemoglobin with effective oxygen-carrying capacity following resuscitation, ATP and phosphocreatine levels in brain tissue failed to recover to baseline values. Simultaneously, the NAD^+^/NADH ratio decreased, indicating a shift in mitochondrial redox status toward a highly reduced state. This reductive pressure sustained the ETC in an over-reduced state, thereby limiting oxidative phosphorylation efficiency and forcing cells to remain dependent on glycolysis for energy production.

It is noteworthy that energy metabolism dysfunction is characterized not only by insufficient intracellular ATP production but also by the abnormal accumulation of extracellular ATP, which has emerged as an important contributor to IRI. In a hepatic IRI model, deficiency of ectonucleoside triphosphate diphosphohydrolase 8 (NTPDase8), an ectonucleotidase specifically expressed by hepatocytes, resulted in markedly elevated ATP levels in both liver tissue and plasma, thereby aggravating liver injury. In contrast, pharmacological inhibition of purinergic P2 receptors significantly attenuated tissue damage (15). These findings suggest that extracellular ATP released following resuscitation functions as a DAMP, activating purinergic signaling pathways and acting in concert with intracellular energy failure to amplify inflammatory responses and tissue injury. However, as these observations were derived from a hepatic IRI model, their relevance to hemorrhagic shock resuscitation-associated IRI remains to be further explored.

Accordingly, in response to the persistent mitochondrial energy metabolism dysfunction observed during hemorrhagic shock and resuscitation, the concept of metabolic resuscitation has emerged in recent years. This strategy aims not only to restore systemic hemodynamics but also to improve mitochondrial function and cellular metabolic capacity (16). Emerging evidence suggests that supplementation with mitochondrial cofactors, such as coenzyme Q10, can enhance mitochondrial function and promote cellular energy metabolism (17). In addition, administration of nicotinamide adenine dinucleotide (NAD^+^) precursors, including nicotinamide mononucleotide, has been shown to restore ETC activity and improve ATP production by maintaining mitochondrial redox homeostasis (9).

Metabolomic studies further support the presence of persistent mitochondrial metabolic dysfunction following hemorrhagic shock resuscitation. Sustained accumulation of lactate and tricarboxylic acid (TCA) cycle intermediates after resuscitation indicates incomplete recovery of mitochondrial oxidative phosphorylation capacity (13). Therefore, therapeutic interventions targeting mitochondrial energy metabolism may facilitate the restoration of oxidative phosphorylation and potentially reverse the persistent metabolic reprogramming associated with hemorrhagic shock resuscitation.

## 3 Excessive oxidative stress

Oxidative stress reaches its peak during the early reperfusion phase and represents a critical driving force that promotes the transition of mitochondria from reversible functional impairment to irreversible structural damage. Excessive mitochondrial reactive oxygen species (ROS) generation not only directly damages mitochondrial components but also initiates subsequent pathological processes, including metabolic dysfunction, mitophagy dysregulation, and mPTP opening. The development of mitochondrial oxidative stress during hemorrhagic shock resuscitation is closely associated with the stage-specific dysregulation of respiratory chain complexes I, II, and III (18, 19). These findings are primarily derived from other ischemia-reperfusion models and therefore represent mechanistic extrapolations that require validation in hemorrhagic shock. During the ischemic phase, disruption of oxidative phosphorylation impairs electron transfer through complexes I and III, resulting in highly reduced states of both NADH and the ubiquinone pool. Concurrently, altered metabolic flux through complex II promotes the reduction of fumarate to succinate, leading to substantial intracellular succinate accumulation. During the early reperfusion phase, rapid oxidation of accumulated succinate by complex II becomes the predominant route for electron re-entry into the ETC. This process further over-reduces the ubiquinone pool and drives reverse electron transport (RET) at complex I, thereby triggering an acute burst of mitochondrial ROS generation (20). During the late reperfusion phase, intracellular succinate is gradually depleted, and RET-mediated ROS production from complex I correspondingly declines. However, persistent structural and functional impairment of the respiratory chain causes complex III, particularly the Qo site, to become a major source of sustained mitochondrial ROS production (18).

During the ischemic phase, Chen et al. (21) demonstrated in an isolated myocardial mitochondrial model that ischemia impaired electron transfer through respiratory chain complexes I and III, resulting in marked reductions of both NADH and the ubiquinone pool. Although mitochondrial ROS production remained relatively limited at this stage, the accumulation of electrons and metabolic substrates established the biochemical basis for the rapid amplification of oxidative stress upon reperfusion. Furthermore, Chouchani et al. (22), using isotope tracing and metabolic flux analyses in murine models of cardiac and systemic ischemia, revealed that succinate accumulation during ischemia primarily originates from fumarate reduction rather than the forward oxidative reaction catalyzed by succinate dehydrogenase. Specifically, during ischemia, aspartate continuously generates fumarate through the malate–aspartate shuttle and the purine nucleotide cycle. Under the highly reduced mitochondrial environment, fumarate is subsequently reduced to succinate via the reverse activity of complex II, leading to substantial succinate accumulation during the ischemic phase. This accumulated succinate then serves as a critical metabolic substrate driving the burst of mitochondrial ROS generation during reperfusion.

During the early reperfusion phase, the rapid restoration of oxygen supply drives accelerated oxidation of succinate accumulated during ischemia, resulting in a massive influx of electrons into the ubiquinone pool and promoting RET at complex I. Under these conditions, the highly reduced ubiquinone pool, together with the restored proton motive force, places complex I in an excessively reduced state, thereby facilitating electron leakage to molecular oxygen and generating large amounts of mitochondrial ROS. Using an *in vivo* ischemia–reperfusion model, Chouchani et al. (22) demonstrated that the sharp increase in ROS production during early reperfusion predominantly originated from complex I by selectively modulating RET activity and monitoring mitochondrial ROS generation. Further mechanistic studies (23) using isolated mitochondria established experimental conditions favoring RET and showed that complex I became the major source of superoxide production only under RET conditions. Collectively, these findings mechanistically confirm the central role of complex I-mediated RET in driving oxidative stress during the early reperfusion phase.

As reperfusion and resuscitation progress, RET at complex I gradually declines because of progressive succinate depletion. However, structural and functional impairment of the ETC persists. Using an isolated mitochondrial model to simulate post-ischemic respiratory chain dysfunction, Chen et al. (21) evaluated ROS generation under reoxygenation conditions and demonstrated that when electron transfer was restricted or the ubiquinone pool remained highly reduced, complex III—particularly the Qo site—became the predominant source of superoxide production. Moreover, under sustained oxygen supply, complex III was capable of continuously generating ROS. These findings suggest that although complex I-mediated RET dominates oxidative stress during early reperfusion, complex III may subsequently become the major source of persistent mitochondrial ROS production during the late reperfusion phase. Although these findings were obtained from cardiac and other ischemia-reperfusion models, they provide mechanistic insights that may be relevant to hemorrhagic shock resuscitation-associated IRI.

Excessive mitochondrial ROS production not only directly damages intracellular macromolecules but also serves as a critical mediator linking mitochondrial injury to systemic microcirculatory dysfunction. Previous studies have demonstrated that, in a model of hemorrhagic shock combined with subrenal aortic occlusion, reperfusion rather than ischemia induced marked shedding of the vascular endothelial glycocalyx, a process largely driven by mitochondrial ROS generation. Notably, treatment with the mitochondria-targeted antioxidant MitoTEMPOL effectively preserved endothelial glycocalyx integrity and attenuated endothelial injury. These findings highlight the pivotal role of mitochondrial oxidative stress in bridging cellular damage and systemic microvascular dysfunction during IRI (24). Accordingly, therapeutic strategies targeting mitochondrial oxidative stress primarily focus on limiting excessive mitochondrial ROS generation and attenuating downstream oxidative injury. These approaches include the use of mitochondria-targeted antioxidants, such as MitoQ, to selectively scavenge mitochondrial ROS (25), as well as interventions aimed at correcting ETC dysfunction or inhibiting pathological RET to reduce ROS production at its source (23). Collectively, these strategies may mitigate oxidative stress–mediated tissue injury and contribute to organ protection during hemorrhagic shock resuscitation.

## 4 Mitochondrial dynamics dysregulation

Mitochondrial dynamics refers to the highly coordinated processes through which mitochondria continuously remodel their morphology and network architecture via fusion and fission. This dynamic balance is essential for maintaining mitochondrial homeostasis, energy metabolism, and mitochondrial quality control (26). During ischemia–reperfusion, pathological factors such as ATP depletion, excessive oxidative stress, and calcium overload disrupt the tightly regulated balance between mitochondrial fusion and fission, leading to mitochondrial fragmentation and network disintegration. These alterations exacerbate mitochondrial dysfunction and drive the progression of IRI (27).

During the ischemic phase, alterations in mitochondrial dynamics are primarily characterized by impaired mitochondrial fusion. Evidence from cardiac ischemia models provides mechanistic insights into these changes, although their specific relevance to hemorrhagic shock remains to be established. Under conditions of hemorrhagic shock–induced ischemia, inadequate oxygen delivery and cellular energy depletion disrupt the mitochondrial fusion process, causing the mitochondrial network to shift from interconnected tubular structures to shorter, dispersed morphologies, thereby reducing fusion capacity. Chen et al. (21) demonstrated in isolated cardiac mitochondria that ischemia markedly impaired ETC activity and was accompanied by loss of mitochondrial structural integrity, providing both functional and structural bases for impaired mitochondrial fusion. Although overt mitochondrial fragmentation is not yet prominent during this stage, mitochondrial network stability and intrinsic self-repair capacity are already substantially compromised.

During the early reperfusion phase, excessive mitochondrial fission is the hallmark of mitochondrial dynamics dysregulation. In cellular and animal models of myocardial ischemia–reperfusion injury, Ong et al. (28). demonstrated that reperfusion markedly increased the translocation of dynamin-related protein 1 (Drp1) to the outer mitochondrial membrane, accompanied by the rapid transformation of mitochondria from elongated tubular networks into fragmented structures. In contrast, pharmacological inhibition of Drp1 significantly improved mitochondrial morphology and attenuated tissue injury. In a rat model of hemorrhagic shock aggravated by seawater immersion, Liu et al. demonstrated that Drp1 was activated and translocated from the cytoplasm to mitochondria in the heart, resulting in excessive mitochondrial fragmentation (29). These findings suggest that mitochondrial fission is rapidly activated and becomes the predominant process during the early reperfusion phase, whereas mitochondrial fusion fails to recover concomitantly, ultimately resulting in profound disruption of mitochondrial network integrity.

During the late reperfusion phase, mitochondrial dynamics dysregulation exhibits persistent pathological features. In a review of experimental studies on myocardial ischemia–reperfusion injury, Rosdah et al. (27). reported that mitochondrial ETC dysfunction persists throughout reperfusion, whereas mitochondrial ROS production fails to return to baseline levels. Concurrently, fission-related signaling pathways remain activated, suggesting that mitochondrial dynamics dysregulation is sustained during the late reperfusion phase and may contribute to ongoing mitochondrial and tissue injury.

Mitochondrial fragmentation resulting from dysregulated mitochondrial dynamics not only compromises cellular energy production but also profoundly influences the initiation and efficiency of mitophagy through alterations in ΔΨm and related signaling pathways. Excessive mitochondrial fission mediated by Drp1 promotes mitochondrial network fragmentation and exacerbates mitochondrial dysfunction, whereas inhibition of Drp1 activity has been shown to significantly reduce mitochondrial fragmentation, preserve mitochondrial function, and alleviate IRI (28, 30). Drp1 targeted strategies, including Mdivi-1 and the Drp1-Fis1 inhibitor/P110, may attenuate excessive mitochondrial fission and mitigate ischemia–reperfusion injury (31, 32). However, Mdivi-1 lacks sufficient Drp1 specificity and can inhibit mitochondrial complex I, whereas P110 more selectively disrupts the pathological Drp1-Fis1 interaction, highlighting the need for cautious interpretation and further validation (33). In contrast, upregulation of mitochondrial fusion-related proteins, including mitofusin 1/2 and optic atrophy protein 1, contributes to the restoration of mitochondrial network integrity and improves oxidative phosphorylation efficiency (34, 35). Overall, evidence from hemorrhagic shock models supports a role for Drp1-mediated mitochondrial fission, while some stage-specific mechanistic evidence is derived from other ischemia–reperfusion models and should therefore be interpreted cautiously.

## 5 Defective mitophagy

Mitophagy is a selective quality control mechanism through which cells eliminate damaged or dysfunctional mitochondria via the autophagy–lysosomal pathway, thereby maintaining mitochondrial homeostasis and cellular energy metabolism (36). During hemorrhagic shock–associated IRI, mitophagy undergoes dynamic stage-specific changes. During the ischemic phase and early reperfusion phase, moderate activation of mitophagy exerts compensatory and protective effects by facilitating the clearance of damaged mitochondria. However, during late reperfusion, persistent or dysregulated mitophagy may further aggravate mitochondrial injury and contribute to the transition from structural damage to inflammatory amplification. A central mechanism underlying mitophagy is mediated by the PTEN-induced kinase 1 (PINK1)/Parkin RBR E3 ubiquitin protein ligase(Parkin) signaling pathway. Upon the loss of ΔΨm, PINK1 accumulates on the outer mitochondrial membrane and recruits the E3 ubiquitin ligase Parkin, which subsequently ubiquitinates multiple outer mitochondrial membrane proteins. These ubiquitinated proteins are recognized by autophagic adaptor proteins, such as sequestosome 1 (p62), which interact with microtubule-associated protein 1 light chain 3 (LC3), thereby facilitating the sequestration of damaged mitochondria into autophagosomes and initiating selective mitophagy (37). In addition to the PINK1/Parkin pathway, receptor-mediated mitophagy also plays an important regulatory role during ischemia-reperfusion injury. BCL2 interacting protein 3 (BNIP3), an outer mitochondrial membrane protein, contains a conserved LC3-interacting region that enables direct binding to LC3 and promotes receptor-mediated mitophagy. Under hypoxic conditions, BNIP3 expression is markedly upregulated by hypoxia-inducible factor-1α, thereby enhancing mitophagic activity (38).

During hemorrhagic shock resuscitation–associated IRI, mitophagy undergoes dynamic stage-specific changes. During the ischemic phase, loss of ΔΨm together with persistent hypoxic signaling activates the PINK1/Parkin pathway as well as receptor-mediated mitophagy pathways involving BNIP3 and FUN14 domain-containing protein 1 (FUNDC1) (37). During the early reperfusion phase, excessive oxidative stress and the burst of mitochondrial ROS generation further enhance mitophagic activity in an attempt to eliminate damaged mitochondria and maintain mitochondrial quality control (39). However, during the late reperfusion phase, sustained mitophagy dysregulation, manifested as either excessive activation or impaired mitophagic flux, may disrupt cellular energy metabolism, aggravate mitochondrial dysfunction, and ultimately contribute to progressive cellular injury and organ dysfunction (40). Some of these mechanisms have been investigated in cellular and non-hemorrhagic ischemia–reperfusion models, and their relevance to hemorrhagic shock should therefore be interpreted cautiously.

During the ischemic phase, insufficient oxygen supply and disruption of the mitochondrial ETC lead to a decline in ΔΨm, thereby triggering accumulation of PINK1 on the outer mitochondrial membrane. Using carbonyl cyanide m-chlorophenylhydrazone (CCCP)-induced mitochondrial depolarization in SH-SY5Y cells, Geisler et al. (41) demonstrated that reductions in ΔΨm markedly increased the association between PINK1 and Parkin in immune precipitates, indicating activation of PINK1/Parkin-mediated mitophagy. Simultaneously, hypoxia-responsive signaling pathways further contribute to mitophagy activation during the ischemic phase. Zhang et al. (38) reported that hypoxia-inducible factor-1αupregulates the expression of BNIP3. Through its LC3-interacting region, BNIP3 directly mediates receptor-mediated mitophagy, suggesting an important role for BNIP3 in hypoxia-induced mitochondrial quality control. In addition, Chen et al. (42) demonstrated in animal models of hypoxia that hypoxia induces dephosphorylation of FUNDC1 and enhances its interaction with LC3, thereby promoting FUNDC1-mediated mitophagy. During the ischemic phase, mitophagy predominantly exhibits an adaptive and compensatory pattern of activation, facilitating the clearance of initially damaged mitochondria and contributing to the maintenance of mitochondrial homeostasis (43).

During the early reperfusion phase, restoration of oxygen supply and metabolic substrates triggers a burst of mitochondrial ROS generation, which further activates the PINK1/Parkin signaling pathway and enhances mitophagy, thereby protecting cells by clearing damaged mitochondria. In a cerebral ischemia–reperfusion model, Lan et al. (44) demonstrated that reperfusion significantly increased the expression levels of PINK1, Parkin, p62, and LC3-II, accompanied by accumulation of autophagy-related markers and formation of autophagosomes, indicating robust activation of mitophagy. Further evidence from a hepatic ischemia–reperfusion model showed that PINK1 overexpression enhanced mitophagic activity while suppressing NLRP3 inflammasome activation, thereby attenuating inflammatory injury (45). In addition, Jiang et al. (46) reported in a pulmonary ischemia–reperfusion model that adiponectin exerted organ-protective effects by activating the SIRT1–PINK1 signaling pathway, which promoted mitophagy and subsequently reduced mitochondrial ROS production and apoptosis. These findings suggest that moderate activation of mitophagy during the early reperfusion phase may function as an adaptive protective mechanism that limits mitochondrial damage and restrains excessive inflammatory responses.

During the late reperfusion phase, mitophagy dysregulation contributes to the progression of cellular injury. Persistent oxidative stress, together with lysosomal dysfunction, may result in either impaired mitophagic flux or excessive mitophagy activation, both of which disrupt mitochondrial quality control and aggravate cellular damage. In a traumatic hemorrhagic shock model, MKK3 depletion reduced PINK1/Parkin-mediated mitophagy, enhanced SIRT1/PGC-1α-associated mitochondrial biogenesis, and restored intestinal mitochondrial function, suggesting that excessive mitophagy may contribute to mitochondrial dysfunction after hemorrhagic shock (47). Lan et al. (44) further demonstrated that excessive activation of PINK1/Parkin-mediated mitophagy during specific stages of ischemia–reperfusion may promote apoptosis rather than confer cytoprotective effects. Moreover, the interplay between mitophagy dysregulation and other forms of regulated cell death should not be overlooked. For instance, in hemorrhage-induced hepatic ischemia–reperfusion injury, ferroptosis has been shown to induce mitochondrial damage and promote neutrophil extracellular trap formation. The E3 ubiquitin ligase TRIM21 may contribute to this process by regulating the stability of the ferroptosis-related protein SLC7A11. These findings suggest that failure of mitochondrial quality control mechanisms may synergize with multiple cell death signaling pathways, ultimately exacerbating tissue injury and amplifying inflammatory responses (48).

During the ischemic phase and early reperfusion phase, appropriate activation of mitophagy facilitates the clearance of damaged mitochondria and contributes to the maintenance of mitochondrial quality control (49, 50). However, during the late reperfusion phase, mitophagy dysregulation may lead to excessive mitochondrial loss or impaired mitophagic flux, thereby disrupting cellular energy metabolism and aggravating mitochondrial dysfunction (44). Therefore, phase-specific regulation of mitophagy through pathways such as PINK1/Parkin, BNIP3, and FUNDC1 may provide a potential therapeutic approach for reducing organ injury after hemorrhagic shock resuscitation. Maintaining a balance clearance of damaged mitochondria and the preservation of mitochondrial energy production is essential for sustaining mitochondrial homeostasis during ischemia–reperfusion injury. Several compounds have been shown to modulate mitophagy and mitochondrial quality control. Urolithin A (51) promoted mitophagy and facilitated the clearance of damaged mitochondria, spermidine (52) enhanced mitophagy through the PINK1/Parkin-related pathway, and melatonin (53) modulated mitophagy-related proteins, improved mitochondrial quality control, and reduced oxidative stress, thereby preserving mitochondrial function and alleviating ischemia–reperfusion injury. Therefore, timely modulation of mitophagy at different stages of reperfusion, rather than sustained enhancement of mitophagy, may be more beneficial for restoring the selective clearance of damaged mitochondria and maintaining mitochondrial quality control and cellular energy homeostasis.

## 6 mPTP opening

During hemorrhagic shock resuscitation, mPTP opening represents a critical event that drives the transition of mitochondrial injury from reversible functional impairment to irreversible structural damage. During the ischemic phase, progressive intracellular Ca^2+^ accumulation places mitochondria in a highly sensitized state for mPTP opening (54). However, the acidic intracellular environment generated during ischemia partially suppresses mPTP activation and transiently delays catastrophic mitochondrial injury. During the early reperfusion phase, restoration of oxygen delivery promotes excessive ROS generation secondary to mitochondrial ETC dysfunction, while intracellular and mitochondrial Ca^2+^ overload further intensifies. Simultaneously, rapid lactate clearance restores intracellular pH, thereby removing the inhibitory effect of acidosis on mPTP opening (55). Under the combined effects of oxidative stress, calcium overload, and pH normalization, mPTP opening is markedly enhanced during early reperfusion. As reperfusion progresses into the late phase, persistent mPTP opening facilitates the release of mitochondrial components, including mitochondrial DNA (mtDNA), which further amplifies sterile inflammatory responses (56). Collectively, these interconnected pathological processes promote sustained mPTP opening, leading to ΔΨm collapse, impaired ATP production, and the release of mitochondrial contents into the cytoplasm and extracellular space, thereby exacerbating cellular injury and organ dysfunction.

During the ischemic phase of hemorrhagic shock, tissue hypoperfusion impairs mitochondrial oxidative phosphorylation and markedly reduces ATP production, resulting in highly reduced states upstream of ETC complexes I and III (22). Under these conditions, intracellular Ca^2+^ continuously enters mitochondria through the mitochondrial calcium uniporter, leading to progressive accumulation of Ca^2+^ within the mitochondrial matrix. This mitochondrial Ca^2+^ overload increases the sensitivity of the mPTP to calcium stimulation and lowers the threshold required for mPTP opening (57). Nevertheless, ischemia-associated intracellular acidosis (pH approximately 6.5–6.8) exerts an inhibitory effect on mPTP activation, thereby suppressing Ca^2+^-induced pore opening and limiting irreversible mitochondrial injury (58). Consequently, during the ischemic phase, although mitochondrial Ca^2+^ progressively accumulates and mitochondria become highly susceptible to mPTP activation, the pore largely remains closed because of the inhibitory effects of the acidic intracellular environment.

During the early reperfusion phase is a critical time window for inducing sustained mPTP opening. During ischemia, succinate accumulates significantly; at the onset of reperfusion, retrotransmission RET occurs at Complex I, triggering an acute burst of mitochondrial ROS generation at the mitochondrial level (23). Previous studies have shown a close association between RET-dependent superoxide anion production and the collapse of the ΔΨm (59). Increased ROS, in synergy with Ca^2+^ overload, lowers the threshold for mPTP opening (57). Concurrently, rapid pH recovery during the reperfusion phase reverses the inhibitory effect of ischemic acidosis on mPTP opening (58). These three processes occur in close temporal overlap, synergistically driving the mPTP from a transient low-conductance state to a sustained high-conductance open state. Stable mPTP opening leads to mitochondrial swelling, outer membrane rupture, and Cytochrome c(Cyt c) release, thereby activating the mitochondrial apoptosis cascade (60); simultaneously, altered mitochondrial membrane permeability promotes the release of mtDNA into the cytoplasm, where it is recognized by cytosolic DNA-sensing pathways and initiates downstream inflammatory signaling (61). In animal models of pulmonary ischemia–reperfusion injury, which provide mechanistic evidence complementary to hemorrhagic shock models, early reperfusion was accompanied by a significant decrease in ΔΨm and increased cytosolic Cyt c levels. Inhibition of mPTP opening by cyclosporine A or cyclophilin D significantly attenuated these changes (62), highlighting the central role of mPTP as a key regulatory node linking mitochondrial dysfunction, cell death, and inflammatory activation during reperfusion. In a rat model of traumatic hemorrhagic shock, CsA improved mitochondrial function, hemodynamics, and tissue perfusion, while experiments in hypoxia-treated vascular smooth muscle cells showed that CsA inhibited mPTP opening, supporting a role for mPTP in mitochondrial dysfunction and vascular hyporeactivity after traumatic hemorrhagic shock (63). Sevoflurane postconditioning inhibited mPTP opening, preserved mitochondrial membrane potential, and reduced hippocampal neuronal apoptosis, thereby improving spatial learning and memory (64).

During the late reperfusion phase, persistent or recurrent mPTP opening further promotes inflammatory amplification and progression of structural tissue injury. MtDNA released into the cytoplasm during the early reperfusion phase continues to accumulate, while sustained mPTP opening further facilitates mtDNA leakage from damaged mitochondria and enhances activation of downstream inflammatory signaling pathways (65).

Accordingly, stage-specific regulation of mPTP activity may represent an important therapeutic strategy during hemorrhagic shock resuscitation. During the ischemic phase, limiting mitochondrial Ca^2+^ overload and preserving the inhibitory effect of intracellular acidosis on mPTP opening may help prevent premature pore activation (66). During the early reperfusion phase, inhibition of succinate-driven RET and ROS bursts, attenuation of mitochondrial Ca^2+^ overload, or direct blockade of cyclophilin D-dependent mPTP opening using agents such as cyclosporine A may preserve ΔΨm and reduce reperfusion-induced injury (67, 68). During the late reperfusion phase, persistent mPTP opening promotes the release of mitochondrial constituents and amplifies sterile inflammatory responses. Therefore, stabilizing mitochondrial membrane integrity or preventing sustained mPTP opening may reduce mtDNA release, attenuate inflammatory amplification, and improve organ function (69). However, some mechanistic evidence discussed above is derived from cellular and non-hemorrhagic ischemia–reperfusion models, and its relevance to hemorrhagic shock requires further validation.

## 7 Release of mitochondria-derived DAMP and inflammatory amplification

Damaged mitochondria function not only as central regulators of cellular energy metabolism and cell death but also as a major source of endogenous DAMPs, thereby acting as both danger sensors and inflammatory amplifiers during IRI. Among mitochondria-derived DAMPs, mtDNA has emerged as one of the most extensively studied inflammatory mediators (61, 70), but it is not the sole mitochondrial danger signal. Other mitochondrial components, including extracellular ATP, cardiolipin, N-formyl peptides, and potentially Cyt c, can also contribute to inflammatory signaling through distinct pattern-recognition or purinergic receptors (71). Together, these mediators may contribute to a broader, stage-dependent mitochondria-derived danger-signaling network. During the ischemic phase, mitochondrial dysfunction and oxidative stress primarily lead to progressive damage, including mtDNA injury and alterations in mitochondrial membrane integrity and cardiolipin. During the early reperfusion phase, disruption of mitochondrial membrane integrity and mPTP opening facilitate the release or extracellular exposure of mitochondria-associated DAMPs, including mtDNA, ATP, N-formyl peptides, cardiolipin and Cyt c, thereby activating innate immune and inflammatory responses (72). As reperfusion progresses into the late phase, persistent release or exposure of these mitochondrial DAMPs continuously engages downstream inflammatory signaling pathways, resulting in sustained inflammatory amplification that further aggravates endothelial dysfunction, microcirculatory disturbances, and organ injury (61).

During the ischemic phase, persistent hypoxia and increased reducing pressure within the mitochondrial ETC promote excessive ROS generation, thereby inducing oxidative damage to mtDNA and alterations in mitochondrial membranes. Chen et al. (73) demonstrated that even transient cerebral ischemia could reduce mtDNA content, which partially recovered following reperfusion; however, prolonged ischemia resulted in sustained mtDNA depletion, suggesting that mtDNA injury occurs as early as the ischemic stage. At this phase, mtDNA primarily undergoes oxidative modification and structural destabilization within mitochondria, while mitochondrial membrane components, including cardiolipin, may also undergo oxidative modification or redistribution. In contrast, large-scale release of mtDNA and other mitochondria-derived DAMPs into the cytoplasm or extracellular space is likely to remain limited during ischemia.

During the early reperfusion phase, restoration of blood flow triggers a burst of mitochondrial ROS generation and disruption of mitochondrial integrity, promoting the release or exposure of multiple DAMPs associated with mitochondrial injury and their recognition by innate immune sensors. Mechanistic evidence for these processes has also been obtained from non-hemorrhagic ischemia–reperfusion models. Using a murine model of pulmonary ischemia–reperfusion injury, Zhao et al. (74) demonstrated that reperfusion markedly increased cytoplasmic mtDNA levels in lung tissue, leading to activation of the cyclic GMP–AMP synthase–stimulator of interferon genes (cGAS–STING) pathway and promotion of neutrophil extracellular trap formation. Furthermore, Huang et al. (75) showed that inhibition of cGAS significantly attenuated endoplasmic reticulum stress, whereas STING activation exacerbated endoplasmic reticulum stress responses in alveolar type II epithelial cells and was accompanied by enhanced inflammatory responses, oxidative stress, and apoptosis. In addition to mtDNA, extracellular ATP is another DAMP that can be released during ischemia–reperfusion injury (76). Qian et al. (77) demonstrated that ischemia–reperfusion injury enhanced ATP release, thereby activating P2X7 receptors and promoting NLRP3 inflammasome activation in renal tubular epithelial cells. Oxidative damage and disruption of the inner mitochondrial membrane may also promote cardiolipin oxidation and translocation to the outer mitochondrial membrane, where exposed cardiolipin has been implicated in NLRP3 inflammasome activation (78, 79). Moreover, mitochondrial N-formyl peptides released following mitochondrial damage can engage formyl peptide receptors, particularly FPR1, thereby promoting neutrophil chemotaxis and activation (80). Cyt c can also be released from mitochondria following severe membrane disruption and cellular injury and may act as an extracellular DAMP (81, 82). Gouveia et al (83). investigated the inflammatory effects of extracellular Cyt c and found that TLR4 inhibition significantly reduced ROS production in Cyt c primed HL-60 cells, suggesting that extracellular Cyt c may promote inflammatory responses at least partially through TLR4 signaling. Collectively, these findings suggest that the release or exposure of multiple DAMPs during early reperfusion, including mtDNA and other mitochondria-associated DAMPs, may contribute to the initiation of sterile inflammatory responses through multiple innate immune pathways.

During the late reperfusion phase, persistent mitochondrial and cellular injury may sustain the extracellular release or exposure of multiple mitochondria-associated DAMPs, including mtDNA, ATP, mitochondrial N-formyl peptides, cardiolipin and Cyt c, thereby maintaining sterile inflammatory signaling. Using a rat lung transplantation reperfusion model, Ju et al. (84) demonstrated that the cGAS–STING–TBK1–IRF3 signaling pathway was markedly activated following reperfusion. Notably, degradation of extracellular DNA by DNase I significantly suppressed pathway activation and attenuated inflammatory responses (85), suggesting that extracellular mtDNA released during reperfusion contributes to cGAS–STING-mediated inflammatory signaling. Clinical studies further support the pathological role of circulating mtDNA in systemic inflammatory injury. Similarly, Yamanouchi et al. (86) demonstrated that circulating mtDNA levels were markedly increased in patients with trauma and severe sepsis and were associated with the extent of tissue injury and mortality risk, suggesting that extracellular mtDNA functions not only as a mediator of inflammatory amplification but also as a potential prognostic biomarker in critical illness. Beyond mtDNA, extracellular ATP, mitochondrial N-formyl peptides, and cardiolipin may continue to engage purinergic, FPR1, and NLRP3-related signaling, respectively, while Cyt c release may further contribute to mitochondrial-mediated cell death and tissue injury (87–89) (**Figure 2**).

**Figure 2:**
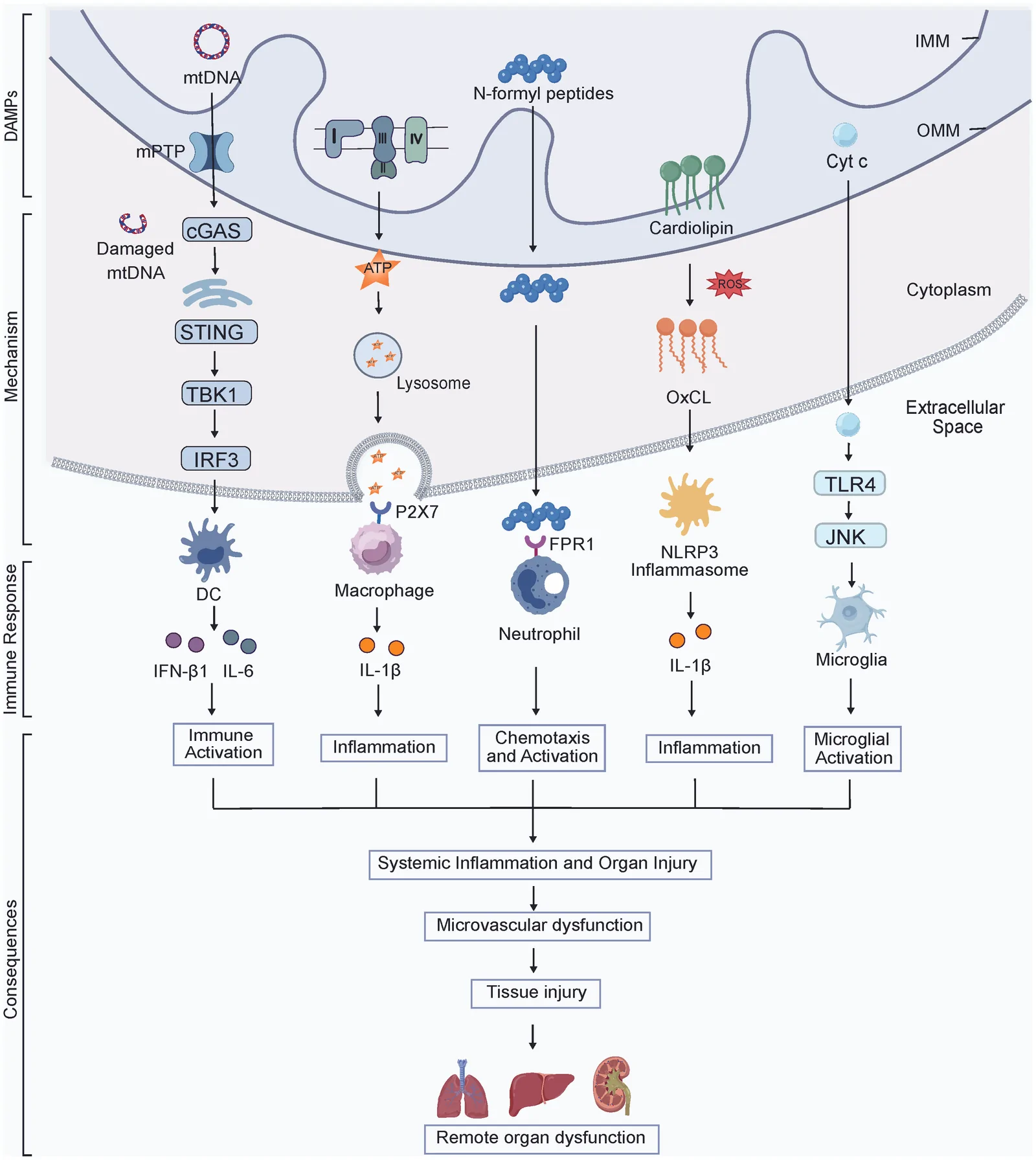
Mechanisms of mitochondrial DAMP signaling and immune activation. Mitochondrial injury is associated with the release or exposure of multiple mitochondrial DAMPs, including damaged mtDNA, ATP, N-formyl peptides, cardiolipin and Cyt c. Damaged mtDNA can activate the cGAS-STING pathway, with downstream activation of TBK1/IRF3, contributing to the production of interferon-β (IFN-β) and interleukin-6 (IL-6). Extracellular ATP can signal through purinergic P2X7 receptors on macrophages and promote inflammasome activation. Mitochondrial N-formyl peptides can engage FPR1 on neutrophils and promote neutrophil chemotaxis, whereas cardiolipin exposure can contribute to NLRP3 inflammasome activation and interleukin-1β (IL-1β) production. Cyt c released from injured mitochondria can activate TLR4 on microglia, leading to JNK activation and microglial activation. These DAMP-associated signaling pathways may converge to amplify inflammatory responses, potentially contributing to systemic inflammation and subsequent multi-organ dysfunction.

These findings suggest that during the systemic inflammatory response associated with hemorrhagic shock resuscitation, multiple mitochondria-associated DAMPs may interact within a complex danger signaling network (70), collectively contributing to innate immune activation, inflammatory amplification, and subsequent organ injury. Accordingly, stage-specific modulation of mitochondria-associated DAMPs, including their release, extracellular accumulation, and downstream recognition, may represent a potential therapeutic strategy during hemorrhagic shock resuscitation (**Figure 3**). Interventions aimed at limiting the release or extracellular accumulation of DAMPs and modulating their associated inflammatory signaling, including cGAS–STING, purinergic, NLRP3 inflammasome, FPR1, and TLR4-related pathways, may help reduce systemic inflammation and secondary organ injury following ischemia–reperfusion. However, some of the mechanistic evidence for individual mitochondrial DAMP and their signaling pathways comes from cellular and non-hemorrhagic ischemia–reperfusion models, and their relevance to hemorrhagic shock remains to be further clarified.

**Figure 3:**
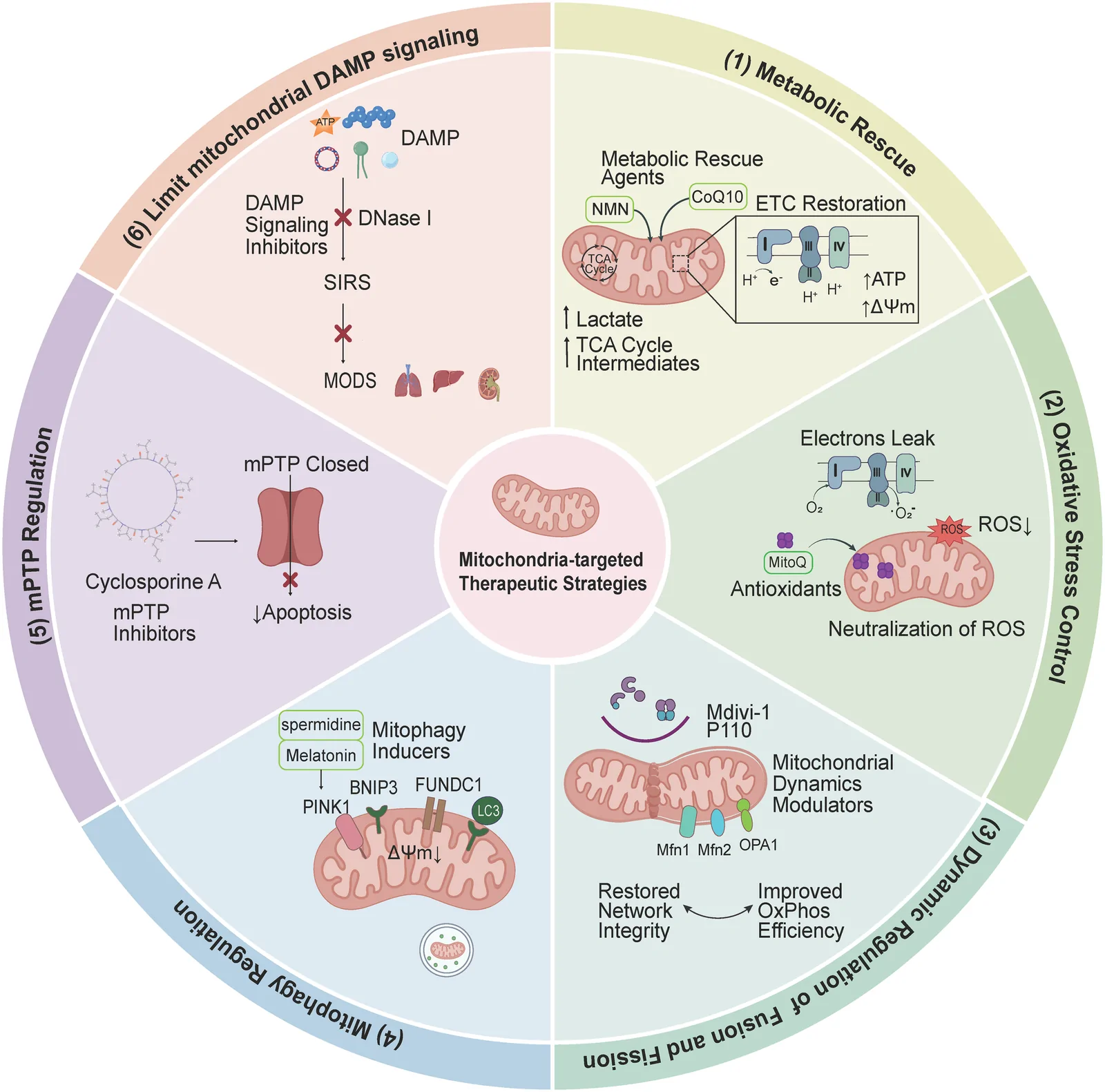
Potential mitochondrial-targeted therapeutic strategies for hemorrhagic shock resuscitation-associated ischemia–reperfusion injury. (1) Metabolic rescue, including coenzyme Q10 (CoQ10) and nicotinamide mononucleotide (NMN), promote electron transport chain restoration and enhance ATP production. (2) Oxidative stress control, represented by MitoQ, reduce mitochondrial ROS generation and limit oxidative damage. (3) Dynamic regulation of fusion and fission through inhibition of excessive Drp1-mediated fission restores mitochondrial network integrity and improves mitochondrial function. (4) Mitophagy regulation, exemplified by spermidine and melatonin, facilitates the removal of damaged mitochondria and supports mitochondrial quality control through PINK1/Parkin-related pathways. (5) Inhibition of mitochondrial permeability transition pore (mPTP) opening by cyclosporine A attenuates mitochondrial dysfunction and apoptosis. (6) Inhibition of mitochondrial DAMP signaling may attenuate downstream systemic inflammatory responses, thereby reducing systemic inflammatory response syndrome (SIRS) and limiting progression toward multiple organ dysfunction syndrome (MODS).

## 8 Conclusion

In summary, mitochondrial homeostasis undergoes dynamic and stage-specific changes during hemorrhagic shock resuscitation. Mitochondrial energy metabolism dysfunction represents an early initiating event, whereas oxidative stress serves as a central driving force that promotes mitochondrial dynamics dysregulation and defective mitophagy. Progressive disruption of mitochondrial quality control ultimately leads to sustained mPTP opening, loss of mitochondrial membrane integrity, and release of mitochondria-derived DAMPs. These events trigger inflammatory signaling pathways and sterile inflammatory responses. These interconnected pathological processes collectively drive the evolution of mitochondrial injury from functional impairment to structural damage and ultimately to inflammatory amplification, contributing to IRI and organ dysfunction.

Although considerable progress has been made in elucidating the underlying mechanisms of mitochondrial injury during hemorrhagic shock resuscitation, the temporal interactions among the different pathological processes and the optimal therapeutic windows for intervention remain incompletely understood. A limitation of the current review is that direct mechanistic evidence from hemorrhagic shock and resuscitation models remains less extensive than that from other ischemia–reperfusion settings. Therefore, some stage-specific patterns discussed here are informed by evidence from myocardial, cerebral, hepatic, and other ischemia–reperfusion models and should be interpreted cautiously, as hemorrhagic shock is characterized by global hypoperfusion rather than regional occlusion, systemic acidosis and hyperlactatemia, coagulopathy, and a reperfusion process whose timing and quality are influenced by the resuscitation strategy. Standardized hemorrhagic shock–resuscitation models and longitudinal assessments are needed to validate these temporal patterns and to better define their applicability to hemorrhagic shock.

The resuscitation strategy may also influence the trajectory of mitochondrial recovery, yet whether contemporary blood-based strategies differentially affect the phase-dependent restoration of mitochondrial homeostasis compared with crystalloid- or colloid-based approaches remains unclear. Future studies should therefore investigate how resuscitation strategies interact with stage-specific mitochondrial responses and determine whether optimizing resuscitation can facilitate mitochondrial recovery beyond the restoration of systemic hemodynamics. Building on these considerations, future research should also focus on developing and clinically validating stage-specific mitochondrial-targeted therapeutic strategies that are tailored to the dynamic progression of mitochondrial injury and integrate metabolic restoration, mitochondrial quality control, and inflammatory regulation.

Importantly, the recognition that mitochondrial injury can generate or expose multiple DAMPs, including mtDNA, ATP, N-formyl peptides, cardiolipin and Cyt c, highlights DAMPs themselves as potential therapeutic targets for limiting sterile inflammation and subsequent organ injury. Given that the release or exposure of different DAMPs may vary with the progression of mitochondrial injury, selectively targeting specific DAMPs or their downstream inflammatory effects at appropriate stages of reperfusion may offer a complementary strategy to mitochondrial protection.

Notably, the clinical translation of mitochondrial-targeted therapies remains challenging. For example, the CIRCUS and CYCLE trials evaluating cyclosporine A, an inhibitor of mitochondrial permeability transition pore opening, in patients with ST-segment elevation myocardial infarction failed to demonstrate clear clinical benefit (90, 91). These findings suggest that the efficacy of mitochondrial-targeted therapies may be influenced by treatment timing, disease context, and the stage of mitochondrial injury. Therefore, further studies are needed to define stage-specific therapeutic windows and determine whether DAMP-directed interventions targeting distinct mitochondrial danger signals can be integrated with mitochondrial-targeted therapies during the dynamic course of hemorrhagic shock resuscitation, which may provide new opportunities for timely organ protection and immune modulation following traumatic hemorrhagic shock.
